# Perspectives of nano-carrier drug delivery systems to overcome cancer drug resistance in the clinics

**DOI:** 10.20517/cdr.2020.59

**Published:** 2021-03-19

**Authors:** Anna Ulldemolins, Joaquin Seras-Franzoso, Fernanda Andrade, Diana Rafael, Ibane Abasolo, Petra Gener, Simo Schwartz Jr

**Affiliations:** ^1^Drug Delivery and Targeting Group, Molecular Biology and Biochemistry Research Centre for Nanomedicine (CIBBIM-Nanomedicine), Vall d’Hebron Institut de Recerca, Universitat Autònoma de Barcelona, Barcelona 08035, Spain.; ^2^Networking Research Centre for Bioengineering, Biomaterials, and Nanomedicine (CIBER-BBN), Instituto de Salud Carlos III, Zaragoza 50009, Spain.

**Keywords:** Drug delivery systems, resistance, nanomedicine, cancer treatment

## Abstract

Advanced cancer is still considered an incurable disease because of its metastatic spread to distal organs and progressive gain of chemoresistance. Even though considerable treatment progress and more effective therapies have been achieved over the past years, recurrence in the long-term and undesired side effects are still the main drawbacks of current clinical protocols. Moreover, a majority of chemotherapeutic drugs are highly hydrophobic and need to be diluted in organic solvents, which cause high toxicity, in order to reach effective therapeutic dose. These limitations of conventional cancer therapies prompted the use of nanomedicine, the medical application of nanotechnology, to provide more effective and safer cancer treatment. Potential of nanomedicines to overcome resistance, ameliorate solubility, improve pharmacological profile, and reduce adverse effects of chemotherapeutical drugs is thus highly regarded. Their use in the clinical setting has increased over the last decade. Among the various existing nanosystems, nanoparticles have the ability to transform conventional medicine by reducing the adverse effects and providing a controlled release of therapeutic agents. Also, their small size facilitates the intracellular uptake. Here, we provide a closer review of clinical prospects and mechanisms of action of nanomedicines to overcome drug resistance. The significance of specific targeting towards cancer cells is debated as well.

## Introduction

According to the World Health Organization, cancer is the second leading cause of death globally, with estimated 9.6 million deaths in 2016. In Europe, 4.2 million new cases were diagnosed and almost 2 million deaths were caused by cancer in 2018^[1]^. Among the different cancers, breast, lung, colon, and prostate cancer caused the most cancer death in 2018. In total, cancer related deaths account for 20% of all deaths in Europe^[2]^. Even though there has been a progress achieved in the development of more effective therapies against cancer, over the past years, recurrence of tumour growth and metastatic spread in the long-term is a common event. Sadly, due to the increased resistance, clinicians have just limited options of effective treatments for secondary tumours. In addition, many classical chemotherapeutic anti-cancer agents kill cancer cells by directly damaging their DNA, which produces high toxicity due to its non-specificity^[3]^. Also, many effective chemotherapeutic drugs are hydrophobic and need to be diluted in an organic solvent (DMSO, Cremophor EL, ethanol *etc*.) that causes toxicity when injected. Therefore, an increment of the therapeutically effective dose is not an option. Consequent drug resistance allows the tumour to grow and spread even with the treatment^[3]^. Cancer resistance can either exist before treatment (intrinsic) or can be generated after therapy (acquired)^[4]^. In addition, heterogeneity among patients and tumours make drug resistance a highly challenging event^[5]^. Closer understanding of the mechanisms involved in drug resistance is needed in order to achieve better outcomes in cancer treatment.

## Causes and mechanisms of cancer resistance

Cancer cells are masters in finding a way to resist the treatment designed to kill them. They may acquire new mechanisms and/or adapt existing mechanisms to protect from the toxic effects of current treatments. Nanotechnology-based drug delivery systems are able to incorporate drugs or gene products with active anti-cancer activity but poor solubility, low bioavailability, or inadequate toxicological profile. In this sense, anti-cancer nanomedicines can improve anti-cancer efficacy mainly by: (1) increasing cytotoxic drug accumulation in tumours thus improving anti-cancer efficacy; (2) prolonging drug systemic circulation, lowering its clearance and decreasing drug accumulation in the normal organs thus reducing undesired toxicity; and (3) deliver different anti-cancer drugs within the same platform. This way higher concentration of chemotherapeutic drugs can be applied, while secondary effects are circumvented. Moreover, multimodal treatment using the same nano-platform could avoid several types of drug resistance (i.e., secondary mutations). Furthermore, nanotechnology-based drug delivery systems (nano-DDS) are able to incorporate drugs or gene products with active anti-cancer activity but poor solubility, low bioavailability, or inadequate toxicological profile. This may lead to an improved efficacy and a superior bioavailability/biodistribution of the carried compound, opening the therapeutic window.

### Elevated drug efflux

**Figure 1:**
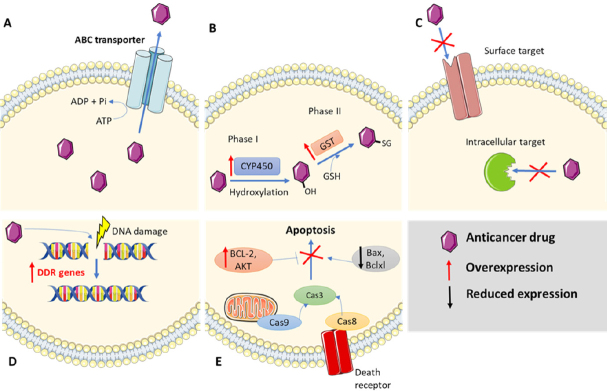
Different mechanisms of cancer drug resistance. A: elevated drug efflux, B: change in the cell metabolism, C: genetic modifications of the drug target, D: enhanced DNA damage response, E: inhibition of apoptosis

Cancer cells may acquire multidrug resistance (MDR), since they express various ATP-binding cassette (ABC) transporter family proteins^[3,5,6]^. When a substrate binds to a transporter, ATP hydrolysis drives a change in conformation that pushes the substrate out of the cell and the concentration of intracellular drug decreases [Figure 1A]^[7]^. ABC transporters (i.e., P-glycoprotein, MRP, BCRP) have a wide substrate specificity and are able to efflux from cells many xenobiotics, including alkaloids, epipodophyllotoxins, anthracyclines, taxanes, and kinase inhibitors^[5]^. Of note, the most chemotherapy resistant tumours express the highest levels of efflux pumps. Importantly, nanomedicine can bypass drug efflux via ABC transporters since it is internalized by endocytosis. This internalization process increases the intracellular accumulation of the drugs and ensures its release in the perinuclear region, avoiding membrane transporters^[8]^. Besides surface functionalization, encapsulation of various active therapeutics in a single nanoparticle platform helps to overcome MDR^[9]^.

Moreover, cells possess a sophisticated mechanism to expel harmful molecules across the plasma membrane. It consists of simultaneous metabolic reactions divided into three phases. Phase I reactions include oxidation, reduction, and hydrolysis of the agents by enzymes that belong to the cytochrome P450 family. In the subsequent phase II, known as consumption and conversion, the main role is played by the glutathione S-transferase (GST) family. They are able to conjugate glutathione (GSH) to a wide range of hydrophobic and electrophilic molecules, making them less toxic, and predisposing them to further modification and being expelled from the cell [Figure 1B]. The conjugate obtained is then actively transported out of the cell by different transmembrane efflux pumps mentioned previously, known as phase III reactions. Notably, cancer cells often profit from adaptation of described mechanisms. Various members of the GST family were found over-expressed in a number of different resistant cancers^[10]^. Identifying patients whose cancer cells over-express a transporter that reduce drug efficacy or has altered expression of other players (GST, P450, GSH) is a plausible approach to determine whether a patient would benefit from nanomedicine based drug delivery^[7]^.

### Genetic alterations

Apart from elevated efflux, some drugs are unable to interact with their molecular target (i.e., EGFR, HER2, topoisomarase II) because it has been altered by means of mutations or modifications [Figure 1C]^[3]^. This leads to a constant battle between generation of new genetic mutations and generation of new inhibitors that restore drug sensitivity. As an example, the first and second generation of tyrosine kinase inhibitors (TKIs), such as erlotinib and gefitinib, have been ineffective in half of the patients with non-small cell lung cancer (NSCLC) due to T790M gatekeeper mutation in EGFR. Moreover, some patients showed resistance as well to third generation of TKIs like osimertinib and rociletinib via C797S mutation. Both mutations impair the binding of TKIs to EGFR in different way^[11,12]^. Hence, a fourth generation of TKI (EAI045) has been designed to overcome both T790M and C797S resistance. EAI045 binds to an allosteric site located in EGFR instead of the modifiable ATP sites^[13]^.

Nanomedicine can in this sense prevent the appearance of secondary mutations via increased dose of inhibitor. Encapsulation of small drug inhibitors and specific targeting may prevent its degradation in the blood stream, accumulation in healthy tissues and thus facilitates higher therapeutic dose while lowering adverse effects.

### Resistance to DNA damage and apoptosis

Inducing DNA damage is a common strategy of many chemotherapeutics to kill cancer cells. The most detrimental DNA damage is the DNA double-strand break (DSB). Nevertheless, the existence of repair pathways described as DNA damage response (DDR) that maintains genomic integrity is well known. It includes DNA double strand break repair, base excision repair, mismatch repair, and nucleotide excision repair^[14]^. Together, they are required due to the constant genomic assault that cells undergo from exogenous sources like ionizing radiation and the action of chemotherapeutic drugs, as well as endogenous sources such as free radicals produced during metabolism due to an aberrant DNA replication. For example, platinum-containing chemotherapy drugs such as Cisplatin cause harmful DNA crosslinks leading to apoptosis, but resistance often arises due to nucleotide excision repair and homologous recombination^[5]^. Therefore, DDR of affected cells to the anti-cancer drugs may result in reduced efficacy of the drugs by DNA lesion repairs, leading to drug resistance [Figure 1D]^[15]^. The inhibition of DNA repair systems are a possible way to sensitize cancer cells to chemotherapeutic drugs and thus to increase their efficacy^[4]^. However, although deregulation of DDR may remit the resistance induced by DNA repair, there is a risk. It may also increase the development of new mutations due to genomic instability^[14]^.

A successful advance in the field was the characterization of poly(ADP-ribose) polymerase (PARP) in patients with germline mutations in the DNA repair genes BRCA1/2. Tumour cells with BRCA1/2 mutation have an impaired DNA DSB repair and they can only be repaired by PARP-mediated base excision repair (BER)^[14]^. In 2014, olaparib became the first PARP inhibitor approved by the Food and Drug Administration (FDA) and the European Medical Agency (EMA) as a treatment for metastatic breast cancer for patients with BRCA1/2 mutation^[14]^. However, its poor water solubility and severe toxicity are two major impediments for the clinical success of olaparib. Encapsulation of olaparib in nano-platform may help to solve both issues. Accordingly, radiosensitization mechanisms and toxicity of olaparib nanoparticles (Ola-NPs) have been already investigated in xenograft mice models. The combination of Ola-NPs and radiotherapy significantly inhibited tumour growth and prolonged survival in mice. Importantly, no additional toxicity caused by Ola-NPs was observed^[9,16]^. This approach of exploiting DNA repair in additional pathways and tumour types opens a new window to overcome drug resistance in cancer.

Another type of cancer cell resistance linked with DNA damage is resistance to apoptosis. In non-cancer cells, if the DNA damage is not repaired, it produces a cell cycle arrest that drives the cell to a programmed cell death known as apoptosis [Figure 1E]^[17]^. Besides, changes in apoptosis-related proteins can also result in drug resistance. For example, tumour suppressor protein p53 (TP53) promotes apoptosis in response to chemotherapeutics, and when it is mutated, drug resistance increases^[5]^.

The capability of nanomaterials to induce non-apoptotic forms of cell death has gained widespread attention in cancer treatment. Different nanomedicines can induce programmed cancer death like paraptosis, overcoming apoptosis based resistance and effectively inhibiting drug resistant tumour growth^[18]^. Also, autophagic cell death induced by nanomaterials alone and as a part of chemo-, radio- and photothermal therapy holds great promise as anti-cancer therapeutic option. Besides, ferroptosis induction by iron-based nanomaterials in drug delivery, immunotherapy, hyperthermia, and imaging systems shows promising results in malignancies^[19]^.

## Epigenetic alterations and cancer heterogeneity

**Figure 2:**
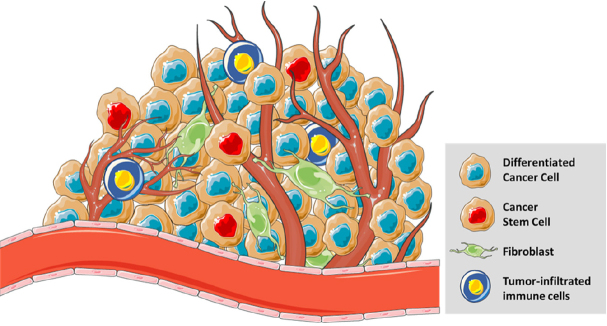
The tumours microenvironment is a heterogenic dynamic entity. It is composed of different cell types (differentiated cancer cells, cancer stem cells, normal stromal cells like fibroblasts, mesenchymal cells, and tumour-infiltrated immune cells). It is localised near the blood vessels to obtain the nutrients needed for its continued growth and survival

The main mechanism that embraces cancer drug resistance phenotypes (including MDR, enhanced DNA repair, and impaired apoptosis) is the epigenetic cancer adaptation^[20-23]^. Epigenetic mutations lead to genomic instability and at the same time generates a great level of genetic heterogeneity within the tumours^[8]^. Therefore, tumours are not a set of homogeneous cancer cells, but they contain various types of cells and extracellular matrixes (ECM) that orchestrate all aspects of cancer hallmarks. Thus, tumours should be considered as a highly complex heterogenic dynamic entity that evolves in time, always trying to adapt and survive in adverse conditions^[24]^. Among the different cell types, particularly involved are normal/differentiated cancer cells, cancer stem cells (CSCs), normal stromal cells, fibroblasts, mesenchymal cells, and tumour-infiltrated immune cells [Figure 2]. In addition, Tumor Micro Environment (TME) also includes several soluble factors such as cytokines and growth factors. All components within cancer cells orchestrate a complex dynamic network with a common objective, survival and spread. The rapid expansion rates of tumours cells trigger several events, such as hypoxia and inflammation, and an adjustment of TME to different contexts. Also, the interaction between cancer cells and neighbouring cells, including stromal cells and immune cells, results in further alterations of the TME cellular components. This crosstalk seems to be performed mainly by tumour-associated fibroblasts and leads to a restructuration of the extracellular matrix and formation of an imperfect vascularization system^[25]^. During tumour growth, cancer cells and TME constituents are continually adapting to the environment conditions, influencing the overall tumour growth^[26]^. Because chemoresistance relies in the clonal evolution generated by mutations and phenotypic variants, cancer cell clones develop resistance to the treatment and remain progressing while current treatment eliminates only the sensitive clones. Indeed, after treatment the overall tumour mass may be reduced, but some remaining resistant clones might survive and eventually cause tumour regrowth and relapse. Resultant recurrent tumours are often defined by a very aggressive tumour phenotypes with very limited treatment options^[24]^.

**Figure 3:**
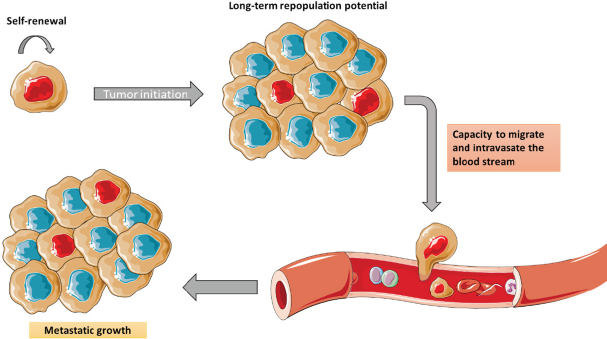
CSCs have stem-like properties. They have self-renewal, tumour initiation capacity, and long-term repopulation potential. CSCs are capable to enter the systemic circulation and generate metastasis. CSCs: cancer stem cells

Indeed, patients with recurrent resistant cancer show higher numbers of cancer stem cells (CSCs) within the tumour. Growing evidence suggest that CSC populations in the TME share several properties of stem cells. There is a direct correlation between cancer recurrence and metastatic growth with the increase of the percentage of CSCs in the tumour. Its importance in oncology relies in the inner capacity of resistance to drugs and toxins through the expression of the different mechanisms described above, such as over-expression of multidrug resistance channels, enhanced DNA repair, impaired apoptosis, and over-expression of detoxifying enzymes like aldehyde dehydrogenase 1 (ALDH1A1) and bleomycin hydrolase (BMLH)^[8,27]^. This subpopulation of cells has stem cell-like properties like self-renewal, tumour initiation capacity, and long-term repopulation potential. Moreover, CSCs are also capable to overcome hypoxic conditions by entering a stable quiescence state and proliferating afterwards. Additionally, these cells survive in non-attachment conditions and can initiate tumour growth *in vivo* leading to an increased capacity to migrate, intravasate, and generate metastasis [Figure 3]^[24]^. Although the total number of CSCs can vary, these features remain constant. It is important to highlight that most of the identified CSC markers are also found in cells with mesenchymal phenotype (e.g., CD44+/CD24-, SPARK, WNT, NOTCH, and ABCG).

To be able to enter systemic circulation and generate metastasis, the cancerous cells need an essential process for tumour progression known as epithelial to mesenchymal transition (EMT). It transforms epithelial cancer cells into a mesenchymal phenotype, promoting local invasion and dissemination to distal organs. This process involves loss of cell-cell adhesion and apical-basal polarity in epithelial cells, followed by a gain in their ability to individually migrate and invade. This conversion correlates with a decrease in epithelial markers (e.g., E-cadherin, cytokeratin, integrin α6β4, laminins, collagen type IV, ZD-1, *etc*.) and an increase in mesenchymal markers (e.g., N-cadherin, vimentin, fibronectin, cadherin-11, integrin α5β1, collagen types I and III, *etc*.)^[24]^.

**Figure 4:**
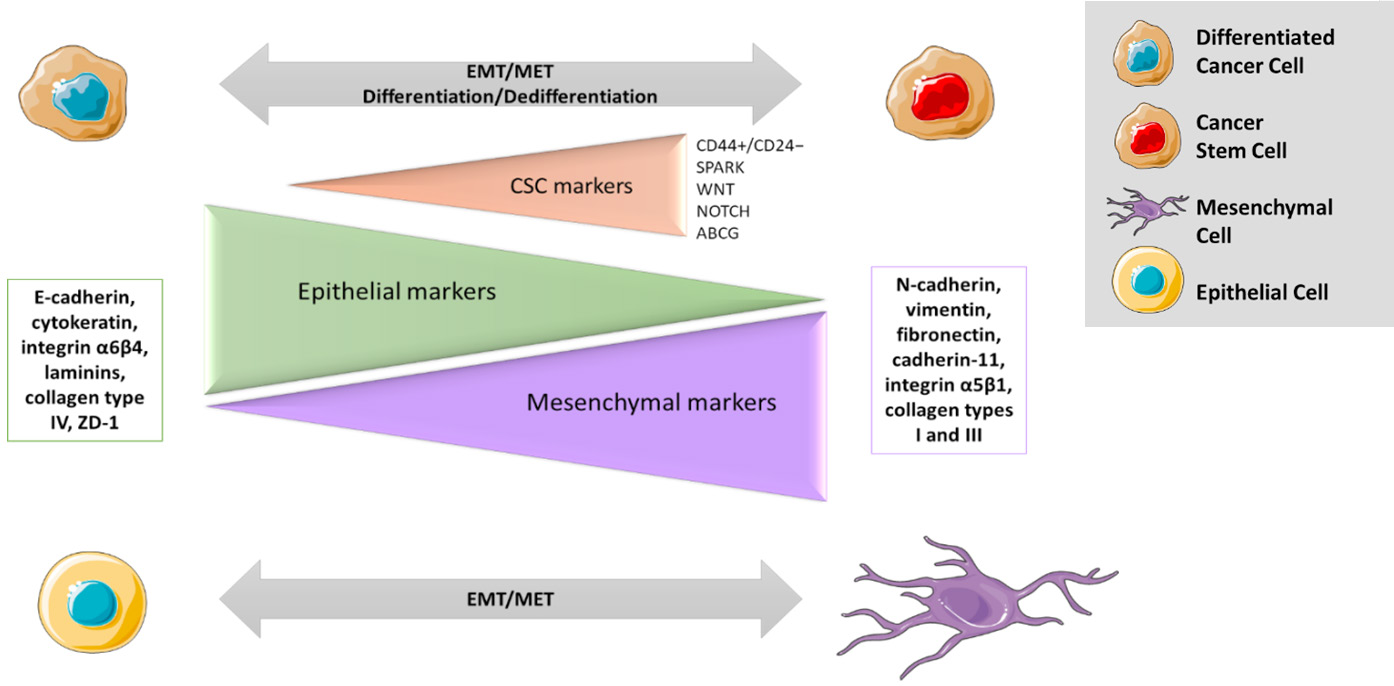
Strong parallels between EMT activation and CSC formation. CSC phenotype is a bidirectional dynamic process. Most of the identified CSC markers are also found in cells with mesenchymal phenotype (e.g., CD44+/CD24-, SPARK, WNT, NOTCH, and ABCG). CSCs: cancer stem cells; EMT: mesenchymal transition

Further, it has been reported that CSC phenotype within a tumour is a bidirectional dynamic progress and there is an equilibrium between CSC and non-CSC populations to maintain a constant level of these cells. For example, IL-6 secreted by non-CSC induces formation of breast CSCs with OCT4 expression. At the same time, OCT4 over-expression is induced via IL-6-JAK1-STAT3 signalling pathway to maintain dynamic equilibrium, reported *in vivo* and in low attachment conditions. Similarly, apoptotic cells excrete prostaglandin PGE2 that promotes proliferation of neighbouring CSCs after chemotherapy, while its neutralizing antibodies (PGE2 Ab) abolish this CSC population [Figure 4]^[8]^.

Nowadays, new strategies for cancer treatment focusing into modulating tumour cell inter-communication and the possibility to modulate the composition of the TME, are being explored. Nanomedicines, dues to its ability to target specific cell types or to deliver multivalent treatment, have gained a lot of attention within the field. Also use of nanomedicine in combination with classic chemotherapy is being explored in order to prevent CSC dynamic phenotype^[28]^.

## Un-druggable targets

Cancer therapeutics interact with their corresponding molecular target to perform their pharmacological actions, which are necessary for the control of disease. Among the different biologic molecules, proteins translated from mRNAs are common targets of therapeutically active small molecules. However, the majority of proteins encoded by the human genome cannot be targeted by conventional therapeutics and therefore, are considered non-druggable^[29]^. Many proteins and enzymes involved in regulatory pathways remain inaccessible because of their cellular location, binding, and/or function (e.g., transcription factors). Alternatives have been screened to generate a therapeutic effect over undruggable targets. One of them is the modulation of signalling pathways using nucleic acid therapeutics (RNA and DNA). However, these therapies reported low transfection efficiency and loss of activity *in vivo* due to their poor stability and rapid degradation^[30]^. Among nucleic acid therapies, RNA molecules have emerged as a new class of therapeutics that may permit the re-targeting of mutated targets, which holds great promise to expand the range of druggable targets, from proteins to RNAs as well as the genome. The use of RNA in gene therapy is increasing due to its unique features compared to DNA. It has transient expression which makes it safer because it does not integrate into the host genome and therefore, there is no risk of insertion mutagenesis^[30]^. Nonetheless, the clinical application of RNAs as a therapeutic tool is limited by its instability and also by its ability to activate immune responses. The design of suitable vehicles for RNA thus results essential for this kind of therapies. Moreover, a vehicle (drug delivery system) may also allow RNA to cross cellular barriers and further, improve blood stability for the molecule^[29]^. In addition to nucleic acid therapies, anti-cancer drugs with poor *in vivo* delivery may also be incorporated in nano-DDS^[8]^.

RNA therapies in cancer treatment can be divided into three categories: those that target nucleic acids (either DNA or RNA), those that target proteins, and those that encode proteins (mRNA). There are two RNA therapies that target nucleic acids which are widely used for cancer treatment: single-stranded antisense oligonucleotides (ASOs), and double-stranded nucleotides that operate through RNA interference (RNAi). The first one consists of a set of nucleotides that prevent mRNA from being translated into protein. They can block the start of translation or mark the mRNA for degradation. RNAi therapies involve small interfering RNAs (siRNAs), and microRNAs (miRNAs), to also degrade mRNA and prevent it from being translated into protein^[29]^. However, these agents are exposed to degradation and various mechanisms of clearance. Therefore, deliver the RNA to the correct place into the correct cells is a great challenge. In order to reach the cells, nanomedicine can be an alternative because nanosystems can protect them from environmental degradation and drive them to target sites^[31]^. Many nanomaterials take advantage of the high negative charge of nucleic acids by complexing them electrostatically with cationic materials. For example, positively charged polymers like poly(l-lysine) (PLL), polyethylenimine (PEI), polyamidoamine (PAMAM), and poly(beta-amino ester)s (PBAEs) can bind nucleic acids into nanoparticles via electrostatic interactions with amines^[31]^. Similarly, cationic lipids have been used recently to encapsulate multiple types of nucleic acids^[30]^. However, to date, no nanomedicine with a gene delivery approach has been approved by the FDA or EMA to treat cancer. Nevertheless, interesting studies have shown substantial promise. An amphiphilic-based gene delivery system that combines pluronic F127 micelles with polyplexes, spontaneously formed between anionic siRNA and cationic PEI by electrostatic interaction^[32]^. The system was loaded with a siRNA against AKT2 which is an important oncogene with a special role in CSC malignancy involved in breast cancer^[33]^. Results showed a significant reduction on cell invasion capacity and strong inhibition of mammosphere formation (i.e., tumour cells able to grow in non-attachment) after treatment, in CSC isolated from MCF-7 and MDA-MB-231 breast cancer cell lines^[32]^. Similarly, RGD-targeted chitosan nanoparticles containing siRNA have been used to successfully downregulate drug efflux transporter P-glycoprotein expression and reverse multidrug resistance in a breast cancer model^[31]^. Furthermore, the first targeted, polymer NP carrying siRNA administered to humans was CALAA-01. It is formulated with cyclodextrin-containing cationic polymer, a PEG-corona, and human transferrin (Tf) as a targeting ligand. This ligand binds to over-expressed transferrin receptors (TfR) on cancer cells and triggers cellular uptake. Its function is to silence the expression of the M2 subunit of ribonucleotide reductase. CALAA-01 is still in human trials with promising results^[34]^. The same targeting ligand has been widely used in other targeted formulations such as SGT-53. It is in phase I and II trials for the treatment of solid tumours, glioblastoma, and metastatic pancreatic cancer. It is a complex composed of a wild type p53 gene [plasmid DNA (pDNA)] encapsulated in a liposome, which is a well-known tumour suppressor gene. Numerous tumours possess a loss or mutation of wild type p53^[5]^. Similarly, SGT 94 with the same platform and targeting ligand as SGT-53, contains RB94 gene (pDNA), a tumour suppressor gene. RB94 gene has shown enhanced tumour suppression and tumour cell killing activity in all tumour cell types studied to date, including bladder cancer cell lines^[35]^.

In this context, ALN-VSP was a novel *first-in-class* liposomal NP-formulated RNAi therapeutic in patients with cancer. It contains siRNAs targeting VEGF and kinesin spindle protein (KSP) and was tested in a phase I clinical trial for patients with advanced solid tumours with liver involvement^[36]^. The promising use of siRNA delivered by liposomal formulations was followed by Atu027. It consists of a liposomal siRNA, which silences the expression of protein kinase N3 in the vascular endothelium^[37]^. A phase I and a phase II clinical trial, Atu027 in combination with gemcitabine, has been performed in patients with advanced solid cancer and advanced metastatic cancer. Also, liposomal-siRNA formulations continued to develop, such as EPHARNA and TKM-080301 with anti-EphA2 and anti-PLK1 siRNA, respectively^[38,39]^. Sadly, the clinical trial of the liposomal micro-ribonucleic acid-34 (miR-34) was withdrawn in phase I due to immune related serious adverse events (NCT02862145).

Finally, regarding inorganic NPs some attempts have also been done to deliver siRNA. An example is NU-0129, composed of siRNA against BCL2L12 bound to gold NPs. This formulation is being investigated in patients with recurrent glioblastoma^[31]^.

## Nano - drug delivery system strategies

Among the various existing nanosystems, nanoparticles (NPs) have the ability to transform conventional medicine by reducing the adverse effects and providing a controlled release of therapeutic agents. There are different requirements in NPs to be used as delivery systems that include size, biocompatibility, and surface chemistry, to prevent unspecific interactions. Ideally, synthesized NPs must remain stable in the blood stream until they reach cancer cells within the TME. They should be able to escape as much as possible from the reticuloendothelial system (RES) and the mononuclear phagocyte system (MPS) to reduce their body clearance. To do so, most systemically administered nanoparticles are greater than 10 nm to prevent premature excretion by kidneys, and below 200 nm to be able to pass through the microcapillaries without producing embolism^[40]^. Besides, their polydispersity index (PDI) value should be close to 0, indicating that batch samples are monodisperse, since even small variations in PDI may cause dramatic changes in biocompatibility and toxicity^[41]^. Cellular uptake and biodistribution is determined also by the surface charge. In general, positive charges facilitate cell internalization compared to neutral and negatively charged nanoparticles but are often more toxic^[42]^. Surface charge also affects their interaction with the biological environment and their binding with blood proteins (via Van der Waals interaction, hydrogen bond, electrostatic force, and hydrostatic interaction) forming a specific protein corona surrounding the nanoparticle. In this context, corona formation might modulate the pattern of hemolysis, thrombocytosis activation, and cellular uptake of the nanoparticles^[40]^. This may be partially solved by the use of poly (ethylene glycol) (PEG) in the surface of the drug-delivery system^[43]^. PEGylation has the ability to enhance NP retention time. Basically, it prolongs the circulation time by increasing its hydrophilicity and reducing the rate of glomerular filtration. Furthermore, it forms a hydrophilic shield that is able of masking the antigenic sites of the proteins and provide protection from reticuloendothelial cells, proteolytic enzymes, and phagocytes. Therefore, it delays recognition by the immune system and increases the chance of NPs to target the desired tissues/cells^[43]^.

The NPs can be made from a variety of materials. To date, they are classified into five categories: lipid-based NPs (liposomes, stealth liposomes, solid lipid nanoparticles (SLNs)], polymer-based NPs [micelles, polymeric nanoparticles, albumin-bound nanoparticles (Nab), dendrimers], inorganic NPs [metallic and metal oxide nanoparticles, silica nanoparticles, carbon nanotubes (CNTs)], drug conjugates (antibody-drug conjugate, polymer-drug conjugate, polymer-protein conjugate), and viral nanoparticles [Table 1]^[44]^. The most used nanoparticles in the clinics for oncology applications are liposomes, polymeric NPs, protein-based NPs, and inorganic NPs.

**Table 1:** Different anti-cancer nanotherapeutic strategies and their advantages and disadvantages

Platform	Types	Advantages	Drawbacks
Lipid based nanocariers	Conventional liposomes	Reduced adverse drug effects	Rapid clearance via RES Toxicity
	Stealth Liposomes (PEGlyated)	Reduced toxicity More circulation times Passive targeting	EPR effect dependent No improved efficacy Toxicity
	Solid Lipid Nanoparticles (SLNs)	More drug capacity Low cost production Easy scale-up Toxicity reduction	Polymorphic transition risk Stability challenges Eventual particle increase
Polymer based nanocarriers	Polymeric Micelles	High drug entrapment Bio-stability	Undefined microstructure Unclear tissue distribution
	Dendrimer	Abundant surface functional groups Monodispersed Long drug retention time Low side effects Convenient usability	Complex preparation process Possible toxicity and immunogenicity Poor biological barrier escape ability
	Nanoparticle Albumin bound (Nab)	Natural carrier of hydrophobic molecules Endocytosis enhanced (gp60 receptor)	Side effects Immunogenic Poor metabolic stability
	Polymeric Nanoparticle	Chemical versatility Complete drug protection High drug-loading capacity Sustained release Good stability Low toxicity Long body circulation Targeting	Limited carrier materials Limited industrial preparation Poor long-term stability Poor effectiveness Poor safety
Drug conjugates	Antibody - Drug	Active targeting Very specific	Coupling strategies Specific targeting necessary
	Polymer - Drug	Tailored biodistribution of drug	Mostly passive targeting
	Polymer - Protein	Clinically used	Immunogenic Poor metabolic stability
Inorganic nanoparticles	Silica Nanoparticles	Inert Safe profile Control of the porous size to introduce drugs Active targeting Low density Large specific surface area High adsorption Unique permeability Favourable optical performance	Toxicity of synthetic process Ambiguous tissue distribution and assembly Potential toxicity
	SPIONs	Unique optoelectrical properties Long-time circulation	Not biodegradable Elimination
	Carbon nanotubes	Excellent optical, electrical, and thermal properties Easy and economical preparation	Single structure Fewer surface modification Potential toxicity
	Gold Nanoparticles	Optoelectrical properties Large specific surface area	Not biodegradable Elimination High cost Aggregation
Viral nanoparticles		Gene therapy High efficacy	Immunogenic Safety problems (Viral spread to unaffected organs) Expensive Limited cargo capacity

RES: reticuloendothelial system; EPR: enhanced permeability and retention

### Lipid-based NPs

Liposomes are frequently used in nanodrug formulations due to their unique properties. They consist of a self-assembling spherical vesicle composed of a lipid bilayer membrane arranged around an empty core that can carry either hydrophilic or hydrophobic compounds. Liposomes can also accumulate at the site of a tumour and deliver higher drug loading. They can also be generated to be temperature- or pH- responsive using lipids of different fatty-acid-chain lengths. This permits the controlled release of their contents only when exposed to specific environmental conditions. However, liposomes have short circulating times due to rapid clearance. This problem has been minimized by PEGylation of the liposome surface as previously mentioned (stealth liposomes). However, toxicity issues such as hypersensitivity reactions have been reported^[45]^.

Recently, SLNs, which are made of solid lipid matrix and a surfactant layer, have gained attention because they present advantages in respect to conventional and stealth liposomes. As an example, encapsulation in SLN of erlotinib (a TKI) and gemcitabine (a chemotherapeutic agent), commonly used in the treatment of NSCLC, have been reported. The formulations showed an improved therapeutic effectiveness and enhanced safety when used against human alveolar adenocarcinoma epithelial A549 cell line^[46,47]^. SLNs have an improved safety profile, high stability, controlled release, easier scale-up and low-cost production. However, they have also limitations because there is a risk of polymorphic transitions that can cause problems in stability, drug leakage, and particle size increase^[48]^.

### Polymer-based NPs

Polymer-based NPs are easily synthesized in a wide range of sizes through organic synthesis methods and are commonly used in nanomedical research. Polymers can be natural, synthetic, or pseudo-synthetic. Polymeric NPs are suitable for controlled release applications, for increasing circulations time and drug half-life, and for increasing biocompatibility and solubility without body accumulation. Nevertheless, there are relevant issues regarding the limited shape and broad size distribution of current formulations. Polymeric NPs are typically spherical, while a wide variety of different sizes may be generated during synthesis. Among the different polymeric approaches, micelles are self-assembling polymeric amphiphilic NPs that can be customized for a slow and controlled delivery. They can achieve different particle size, drug loading, and release characteristics depending on their composition. Micelles have a hydrophobic internal core, which can be used to encapsulate drugs that have poor solubility to allow dissolution in aqueous solutions. Polymeric micelles that encapsulate highly hydrophobic zileuton, a potent inhibitor of CSCs, showed significant reduction of CSCs percentage within tumours and effectively reduced the number of circulating tumour cells (CTCs) in the blood stream and the spread of metastatic cells^[49]^.

Of note, within this NP category, Nab technology emerged when researchers exploited the properties of proteins found in the blood serum that would facilitate transport and dilution of drugs during circulation. In this context, albumin has a number of characteristics that make it an attractive drug carrier and has been particularly used in oncology. Indeed, it is a natural carrier of endogenous hydrophobic molecules such as vitamins and hormones. Therefore, poor water-soluble molecules are attached to albumin in a reversible non-covalent manner^[41]^. The clinical use of Nab NPs currently marketed will be discussed in the next chapter. Further, gemcitabine, a first-line therapy for pancreatic cancer, has been also successfully loaded in human serum albumin NPs and showed strong inhibitory effect on tumour growth against the pancreatic tumour cell line BxPC-3 both *in vitro* and *in vivo*^[50]^.

### Drug - conjugates

Active agents may also be covalently linked to antibodies (antibody-drug conjugates) and to peptides (protein-drug conjugates). Moreover, therapeutic proteins as active agents can also be linked to polymers (polymer-protein conjugate). The conjugates are intended to improve the delivery of drugs into desired tissues or cells without necessarily impacting drug solubility, stability, or biodegradability. In contrast, nanocarriers based on lipids, proteins, glycans, or synthetic polymers, usually encapsulate the drug and therefore avoid the need to covalently link the drug to the carrier^[41]^. Indeed, polymer-drug conjugates are widely used in preclinical studies such as *N*-(2-hydroxypropyl)methacrylamide (HPMA) copolymer-cyclopamine conjugate (P-CYP) against CSCs and HPMA copolymer-docetaxel conjugate (P-DTX) against bulk tumour cells, both tested in prostate cancer cell lines (PC-3)^[51]^.

### Inorganic NPs

In addition to organic NPs, a large number of inorganic materials, such as metal oxides, metal, or silica, can be used to create NPs. Specifically, metal and metal oxide NPs are in the focus for their potential use in therapeutic and imaging applications. Superparamagnetic iron oxide nanoparticles (SPIONs) are gaining attention because they show low toxicity, long circulation times, and are often biodegradable. Also, gold has a unique combination of thermal and optical properties which are relevant to design better theragnostic applications. Properties of gold NPs can be modulated by changes in their physicochemical characteristics (size, shape and surface chemistry). Excitement of electrons in gold NPs by electromagnetic radiation can generate a considerable amount of energy. In fact, colloidal gold NPs act as excellent radiosensitizers when exposed to high-energy electromagnetic radiation^[52]^.

Moreover, carbon nanotubes are carbon cylinders composed of benzene rings that have interesting structural, mechanical, electrical, and optical properties for drug delivery and potential new therapies purposes. For example, the anti-cancer drug combretastatin A4 (CA4) was covalently linked with single-walled carbon nanotubes (SWCNT) with a superior cell cycle arrest than the free CA4^[53]^. Also, silica NPs have emerged as an interesting candidate for anti-cancer therapy owing to its biodegradability, rapid release kinetics, and purpose driven tissue distribution. They are often used in imaging techniques, while their structure and porosity permit the loading of different drugs. For example, doxorubicin delivery via folate-targeted mesoporous silica nanoparticles (MSN) greatly improves the efficacy of the free drug in a xenograft tumour model^[54]^.

### Viral nanoparticles

Due to the inherent infective properties of viruses, many researchers are using tumour-homing viruses engineered to express therapeutic proteins as anti-cancer therapies^[41]^. For example, pox viruses such as myxoma or vaccinia strains, which preferentially replicate in tumour cells due to specific features of cancer cells such as blockage of apoptotic pathways, deregulation of cell replication machinery, and immune evasion. The synthetized pox virus JX- 594 was designed to replicate in tumour cells and destroy them via activation of the EGFR-Ras-MAPK signalling pathway. Unfortunately, despite the remission observed in some patients, flu-like symptoms and hyperbilirubinemia were common side effects^[41]^. Among the different oncolytic viruses currently tested, none have yet reached the market. Their major drawbacks are concerns about biosafety and cytocompatibility^[41]^.

## Current clinical status of drug delivery systems in oncology

### Clinically approved nanomedicines

The first nanoformulated drug approved by the FDA was Doxil [Doxorubicin hydrochloride in PEG coated liposomes (stealth liposomes)] to treat Kaposi’s sarcoma in patients with human immunodeficiency virus (HIV) in 1995 [Table 2]. This approval was mainly based on its low toxicity compared with the conventional drug, in particular, the reduction of cardiotoxicity^[55]^. Nowadays, it is still widely used for its original indication and to treat breast and ovarian cancer, and multiple myeloma as well^[52]^. Furthermore, Abraxane, a formulation of paclitaxel (PTX) complexed with albumin bound nanoparticle was approved by the FDA in 2005 to treat metastatic breast cancer since substantial reduction of toxicity was demonstrated^[56]^. Because of its high hydrophobicity, PTX has to be diluted in polyethoxylated castor oil (Kolliphor EL, formerly known as Cremophor EL) producing strong systemic and neurological toxicity^[57]^. The increased tolerance of Abraxane which does not use Cremophor, implies better safety profile and allows the administration of higher doses of PTX, yielding greater efficacy.

**Table 2:** Clinically approved nanoformulations for oncology in Europe and United States ordered by year of approval

Name	Formulation	Type	Indications	Year
Doxil/Caelyx	PEGylated Liposomal doxorubicin	Liposome	Kaposi sarcoma, ovarian cancer, multiple myeloma	1995 (FDA)
DaunoXome	Liposomal daunorubicin	Liposome	Kaposi sarcoma	1996 (FDA)
DepoCyt	Liposomal cytarabine	Liposome	Lymphoma, leukemia	1999 (FDA)
Myocet	Liposomal doxorubicin	Liposome	Breast cancer	2000 (EMA)
Eligard	Leuprolide acetate and polymer [PLGH (poly (dl-lactide-coglycolide)]	Polymeric nanoparticle	Prostate cancer	2004 (FDA)
Abraxane	Albumin-bound paclitaxel nanoparticle	Albumin-bound nanoparticle	Breast cancer, non-small cell lung cancer, pancreatic cancer	2005 (FDA)
Oncaspar	PEGylated L-asparaginase conjugate	Protein nanoparticle	Acute lymphoblastic leukemia	2006 (FDA)
Ontak	Interleukin (IL)-2 receptor antagonist with diphtheria toxin	Protein nanoparticle	Cutaneous T-cell lymphoma	2008 (FDA)
Mepact	Liposomal mifamurtide	Liposome	Osteogenic sarcoma	2009 (EMA)
NanoTherm	Iron oxide nanoparticles	Metallic nanoparticle	Brain tumours	2011 (EMA)
Sylatron	PEGylated interferon alfa-2b	Protein nanoparticle	Melanoma	2011 (FDA)
Adcetris	CD30- targeted antibody (Brentuximab) and MMAE conjugate	Antibody-drug conjugate	Non-Hodgkin lymphoma	2011 (FDA)
Marqibo	Liposomal vincirstine sulfate	Liposome	Acute lymphoblastic leukemia	2012 (FDA)
Kadcyla	HER2-targeted antibody (Trastuzumab emtansine) and microtubule inhibitor conjugate	Antibody-drug conjugate	HER2-positive, metastatic breast cancer	2013 (FDA)
Onivyde	Liposomal irinotecan	Liposome	Pancreatic cancer	2015 (FDA)
Vyxeos	Liposomal daunorubicin and cytrabine	Liposome	Acute myeloid leukemia (AML)	2017 (FDA)
Apalea	Paclitaxel micellar	Micelle nanoparticle	Ovarian cancer	2018 (EMA)
Hensify	Hafnium oxide nanoparticles	Metallic nanoparticle	Soft tissue sarcoma	2019 (EMA)

As stated earlier, the most well-established polymer used for drug delivery is PEG. It is used in clinically approved polymer-drug formulations like Oncaspar, a PEGylated L-asparaginase to treat acute lymphoblastic leukemia^[58]^ [Table 2]. Furthermore, biodegradable polymers such as PLGH poly(dl-lactide-coglycolide) are used in nanomedicines such Eligard encapsulating leuprolide acetate for the treatment of prostate cancer^[59]^. Also, Apalea is a recently EMA approved micelle formulation of paclitaxel to treat ovarian cancer.

Regarding inorganic nanoparticles, and specifically SPIONs, Nanotherm has been approved by EMA in 2011 [Table 2]. It uses aminosilane-coated SPIONS for local hyperthermia treatment of glioblastoma tumours^[59]^. To achieve intra-tumoural hyperthermia, a magnetic field is applied to heat nanoparticles injected into the tumour. The generated heat is enough to cause programmed and nonprogrammed cell death.

In the same context, gold NPs are also promising as antineoplastic agents either alone or as drug delivery vectors. As mentioned earlier, colloidal gold NPs have been successfully tested against brain tumours as Nanotherm (iron oxide NPs). However, to date, there are no inorganic NPs approved by the FDA or EMA for drug delivery purposes in cancer.

Regarding combination therapy, Vyxeos (CPX-351) was the first dual - drug liposome approved by FDA in 2017 [Table 2]. It is a liposomal NP for the treatment of acute myeloid leukemia that incorporates the drugs cytarabine and daunorubicin in an optimized 5:1 molar ratio^[60]^. Following Vyxeos, a formulation of solid lipid nanoparticles for the co-delivery of paclitaxel and α-tocopherol succinate-cisplatin prodrug has been also developed in order to achieve synergistic antitumour activity against cervical cancer. It exhibited high tumour tissue accumulation, superior antitumour efficiency, and lower *in vivo* toxicity^[61]^.

Preclinical co-delivery studies with polymeric nanoparticles have also been reported. For instance, the co-encapsulation of rapamycin in combination with the chemosensitizer piperine in Poly(D,L-lactide-co-glycolide) (PLGA) NPs. Piperine is known to be a P-glycoprotein (ABCB1) inhibitor, one of the most studied MDR channels mentioned in the previous chapter. The formulation showed an improved bioavailability and efficacy in the treatment of breast cancer^[62]^. Similarly, it has been proposed the use of PLGA-PEG-PLGA NPs to co-encapsulate 5-FU and Chrysin, a natural compound known to enhance the therapeutic efficacy of chemotherapy in colon cancer HT29 human cell line. NPs loaded with both 5-FU and Chrysin were found to have significantly higher growth inhibitory effect^[63]^.

### Nanomedicines in clinical trials

Nanoparticles are in constant development to improve the current treatments and provide better clinical outcomes. Increasing numbers of clinical trials are taking place [Table 3], many of them focused on liposomal formulations. One of them is Promitil (PL-MLP), a pegylated liposomal formulation of Mitomycin C, a highly toxic drug for the treatment of anal squamous cell carcinoma. In phase Ia/b study in metastatic CRC, PL-MLP treatment results in a substantial rate of disease stabilization and prolonged survival in patients achieving stable disease^[64]^. Another one is Thermodox which consists of a liposome-bound doxorubicin formulated with thermally sensitive lipids. There is a disruption of the lipid bilayer when the lipids are exposed to high heat. There are several clinical trials combining Thermodox with radiofrequency ablation in hepatobiliary tumours and most recently, in breast cancer. Furthermore, a liposomal SN-38, an active metabolite of the topoisomerase inhibitor irinotecan, is also being studied in metastatic colorectal cancer^[52]^.

**Table 3:** Nanoformulations for cancer treatment currently studied in clinical trials without targeting ligands

Name	Type	Formulation	Indications	Phase
Promitil (PL-MLP)	Liposome	PEGylated liposomal mitomycin C	Solid tumour s	Phase I (NCT01705002)
Thermodox		Thermosensitive liposomal doxorubicin	Breast cancer hepatocellular carcinoma	Phase III (NCT00617981)
LE-SN38		Liposomal SN-38	Metastatic colorectal cancer	Phase II (NCT00311610)
SPI-077		Stealth liposomal cisplatin	Platinum-sensitive ovarian cancer	Phase II (NCT00004083)
Docetaxel-PM	Polymeric micelle	Docetaxel Polymeric micelle	Metastatic head and neck squamous cell carcinoma	Phase II (NCT02639858)
Nanoplatin (NC-6004)		mPEG-b-poly (glutamic acid) cisplatin)	Head and neck cancer	Phase I (NCT02817113)
NK012		mPEG-b-poly (glutamic acid) SN38	Small cell lung cancer	Phase II (NCT00951613)
NK105		(mPEG-b-poly (aspartic acid) paclitaxel)	Metastatic or recurrent breast cancer.	Phase III (NCT01644890)
NC-4016		mPEG-b-Poly (glutamic acid) oxaliplatin	Advanced solid tumour s or lymphoma	Phase I (NCT03168035)
NC-6300		mPEG-b-Poly (aspartate-hydrazone) epirubicin	Advanced solid tumours or soft tissue sarcoma	Phase I (NCT03168061)
NC-6004		mPEG-poly(glutamic acid) with cisplatin	Locally advanced or metastatic pancreatic cancer	Phase I/II (NCT00910741)
Opaxio	Polymer - drug conjugated	Polyglutamic acid-conjugated (poliglumex) paclitaxel	Advanced ovarian, peritoneal or fallopian tube cancer	Phase III (NCT00108745)
CRLX101		Poly-β-cyclodextrin-PEG-camptothecin	Non small cell lung cancer	Phase II (NCT01380769)
CRLX301		Poly-β-cyclodextrin-PEG-docataxel	Advanced solid tumours	Phase II (NCT02380677)
EZN-2208		Multi-arm mPEG-SN38 conjugate	Metastatic breast cancer and colorectal carcinoma	Phase II (NCT01036113) (NCT00931840)
XMT-1001		Polyacetal-camptothecin conjugate	Small cell lung cancer and Non small cell lung cancer	Phase I (NCT00455052)
NKTR-102		PEGylated irinotecan	Advanced lung cancer and metastatic breast cancer Relapsed small cell lung cancer	Phase II (NCT02312622) (NCT01876446)
Aurimune	Gold NP	TNFα bound to PEGlyated gold NP	Advanced solid tumours	Phase I (NCT00356980)
ABI-008	Nab	Nanoparticle of albumin-bound docataxel	Metastatic breast cancer, prostate cancer	Phase II (NCT00531271)
ABI-009		Nanoparticle of albumin-bound rapamycin	Solid tumours, bladder cancer	Phase I/II (NCT00635284)
ABI-011		Nanoparticle of albumin-bound Thiocolchicine dimer	Solid tumours, lymphoma	Phase I/II (NCT01163071)

Regarding polymeric nanoparticles, Opaxio is one of the most promising nanomedicines. It contains polyglutamic acid-conjugated (poliglumex) paclitaxel. It has shown potential in the treatment of ovarian cancer. A randomized phase III study in women with advanced ovarian, primary peritoneal, or fallopian tube cancer is undergoing (NCT00108745). CRLX101 is a polymer - drug conjugate formulation of camptothecin, a topoisomerase I inhibitor, and a cyclodextran-PEG polymer which is being studied alone and in combination with other drugs in numerous phase I and II clinical trials in the treatment of lung cancers, gynecological malignancies, and solid tumours^[65]^. Recently, a phase II trial has successfully concluded in NSCLC (NCT01380769). In addition, the same polymer conjugated with docetaxel named CRLX301 is also being studied in a phase I/II clinical trial in patients with advanced solid tumours (NCT02380677). Furthermore, a micellar formulation of cisplatin (Nanoplatin, NC-6004), is being investigated in several phase I clinical trials studying its use alone or in combination with other chemotherapies.

Regarding the inorganic NPs, as mentioned previously, non of the gold NPs have reached the market so far. Nonetheless, they have shown promise as antineoplastic agents. For example, gold NPs have been studied in a phase I clinical trial as a drug delivery vector of the toxic antitumour agent tumour necrosis factor alpha (TNFα) (Aurimune, CYT-6091) constituted by a recombinant human TNFα attached to gold NPs using a PEG linker (NCT00356980).

### The relative importance of targeting

**Figure 5:**
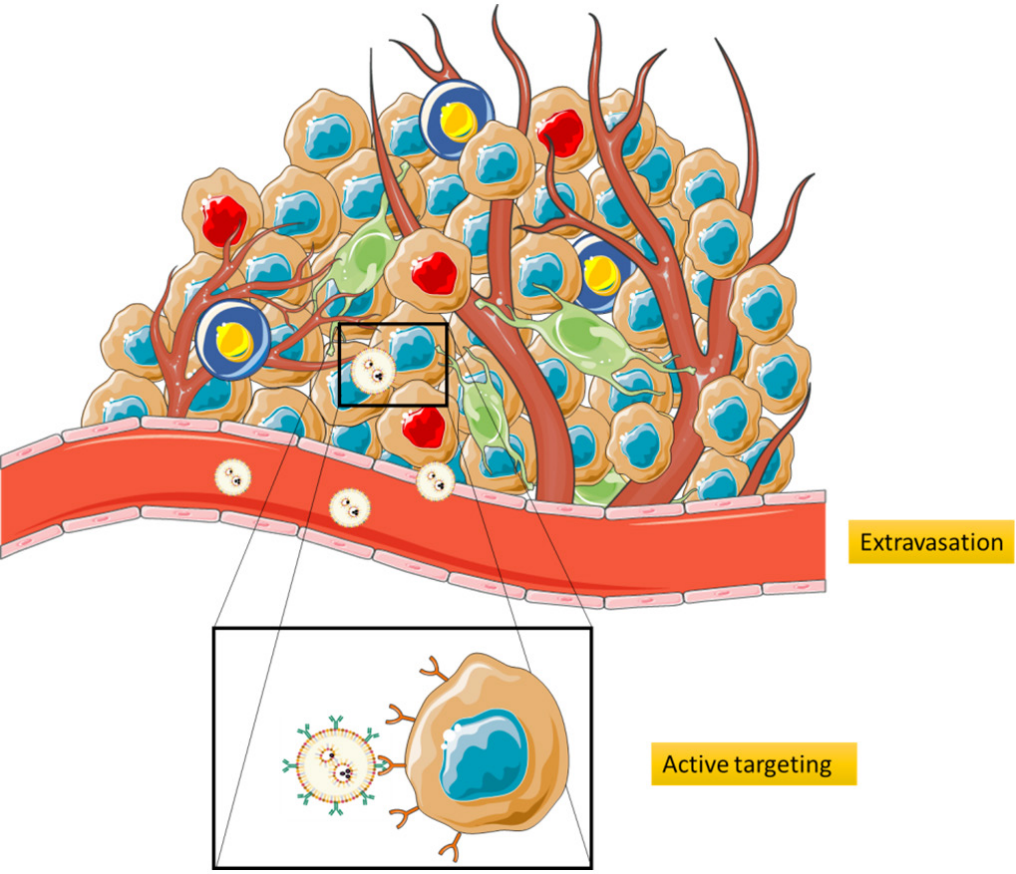
Extravasation and cell targeting. The abnormally wide fenestrations in the blood vessels and the lack of lymphatic drainage facilitates extravasation of NPs. Once in the tumour micro environment (TME), the targeting moiety of the NPs enable its interaction with the desired cells, providing active targeting. NPs: nanoparticles

A majority of nanoparticles [Tables 2 and 3] are passively accumulated at the tumour site. Passive targeting is known as the non-specific accumulation of the NPs in the cancer tissue. It is especially applicable to solid cancers where there are increased blood vessel and transporter permeations, and retention of nanomedicines [enhanced permeability and retention effect (EPR effect)]^[66]^. This effect relies on the specific pathophysiological characteristics of the tumour vessels generated by angiogenesis to provide nutrients to the malignant cells. The abnormally wide fenestrations found in these blood vessels due to the production of vascular permeability factors facilitate the extravasation of NPs^[67]^. The lack or defective lymphatic drainage that characterizes tumour sites also facilitates NPs extravasation [Figure 5]^[68]^. However, there are evidences that show a great variability in the EPR effect among patients and tumour types due to tumour heterogeneity and differences in vascular permeability^[69,70]^.

After the approval of Abraxane and the prominent use of antibody-drug conjugates in the clinics, researchers nowadays tend to use engineered complex targeted particles instead of unmodified proteins. Tumour cells over-express a wide range of molecules in their membrane that can bind to different antigens and regulate tumourigenic pathways, such as angiogenesis or growth metabolic pathways. It is fundamental for an active targeting to use NPs conjugated to a targeting moiety that binds to the surface of specific cell types, such as tumour cells [Figure 5]^[71]^.

One of the main challenges in the field is the engineering of targeted DDS capable of specifically binding cancer cells and avoiding non-cancerous ones. The principal factors that control this targeting are the surface functionalization on the NPs, their physicochemical properties, the specificity of the targeting moiety, and the pathophysiological characteristics of the tumour microenvironment (TME). Indeed, NPs interact with the host environment including a great variety of cells, substrates, and other molecules. This interplay could limit their use in specific applications. However, these drawbacks can be manageable by functionalizing the NPs^[72]^. Through surface modification, the NPs properties can be enhanced. The chemistry behind this functionalization determines the interaction of the NPs with the biological environment^[73]^. Using different strategies, nanoparticles can be functionalized with a variety of ligands such as small molecules, surfactants, dendrimers, polymers, and biomolecules^[74]^. Functionalization of NPs involves conjugation of molecules to the surface of the particles that can be performed by different approaches. Conjugation can be done through noncovalent interactions by attaching specific ligands through affinity-based systems. They include electrostatic interaction, π-π stacking, and entrapping biomolecules in biocompatible films like phospholipids, polymer, and more^[73]^. As an example, it has been reported that tRNA molecules can be noncovalently attached to chitosan NPs through G-C and A-U base-pair electrostatic interactions^[73]^.

Furthermore, it is also possible to perform a direct binding of the molecule of interest to the desired ligands on the NPs surface by covalent conjugation. This approach involves linkage reaction supported by a catalyst. Covalent linkers can be used to form poly(d,l-lactic-co-glycolic acid) (PLGA) - siRNA conjugates for efficient release of siRNA molecules^[73]^. As stated earlier, some NPs improve their capacities by coating the surface with PEG polymer. This coating can also introduce functional groups. Coupling targeting moieties on PEGylated NPs enhances the targeting of tumour cells^[74]^. In this regard, surface coating confers additional functionality to the NPs, and it allows targeting as well. NPs can be coated with organic (monomer and polymer) and inorganic (metal and oxides) layers^[73]^. The different functionalization approaches provide a wide range of potential surface modifications of NPs to enhance their capacities and molecular biological applications^[74]^.

The conjugation process is very complicated because it is important to keep the efficiency of the system unchanged. However, the conjugation can modify the physicochemical properties of NPs, such as size, shape, and others. Other physicochemical properties to take into account are density, orientation, or charge of ligands^[72]^. They play a key role in cellular uptake because the multiple interactions of NPs to target a specific cell membrane induce agglomeration of receptors that facilitate endocytosis. Therefore, optimal parameters vary depending on the size, shape, and material composition of the particle as well as the chemistry of the particular target ligand^[31]^. Independent of the conjugation method used, the functionalization of a NP with a ligand can facilitate binding to a biomarker specifically over-expressed in targeted cells. Among the different ligands, the most used are antibodies, proteins, peptides, small molecules, and aptamers.

Even though the use of active targeting does not seem to be able to significantly change the accumulation of NPs by EPR effect at the tumour sites *in vivo*, the interaction between a ligand and a receptor can be used to improve the internalization of the nanomedicine in tumour cells at the tumour site^[75]^. As described above, beyond the function in the identification of the malignant cells, active targeting (also known as ligand-mediated targeting) facilitates cellular uptake as a result of receptor-mediated endocytosis^[71]^.

Because receptor-mediated endocytosis helps NPs avoid MDR complexes, active targeting might improve cell internalization and therefore, help to overcome drug resistance of metastatic cancers. Thus, there are many nanoformulations with active targeting being studied with the purpose of reducing drug resistance while increasing the effective amount of drug delivered to the tumour cell [Table 4]. Accordingly, the first targeted nanomedicine to enter clinical trials was MCC-465, a liposomal formulation of doxorubicin. It is labelled with a F(abʹ)2 fragment of anti-human MYH14 monoclonal antibody (GAH) that allows cancerous stomach tissues to be specifically targeted. It showed promising results in phase I trials, but did not further progress due to the loss of funding^[35]^. Additional liposomal nanoformulations, such as 2B3-101 and MBP-426, are currently undergoing clinical evaluation for brain and solid tumour treatments, respectively. The first one targets the glutathione transporter and the second one the transferrin receptor^[78]^. Anti-EGFR ILs-DOX is another liposomal NP which is loaded with doxorubicin and has an antibody against EGFR as a targeting ligand and is being studied in patients with gliomas (NCT03603379). Also, a phase II study in patients with metastatic castration-resistant prostate cancer (NCT01812746), was performed with the targeted polymeric nanoparticle BIND014, a docetaxel encapsulated PLGA-PEG NPs targeted to prostate-specific membrane antigen (PSMA). The study outcomes showed reductions in prostate specific antigen (PSA) related to cancer, radiographically confirmed disease control in bone and visceral metastatic disease, favourable CTC conversions, and an acceptable adverse effect profile^[79]^. However, the response rate was lower than expected. Because of this, a randomized phase III compared with the standard docetaxel, which is widely used and effective, will be highly challenging. Moreover, the role of PSMA is still not well elucidated and would require additional studies. Due to the importance of targeting cancer resistance mechanisms, a p-glycoprotein targeting micellar formulation of doxorubicin (SP1049-C) is undergoing clinical evaluation. It is currently in phase II to treat patients with advanced adenocarcinoma of the oesophagus^[76]^.

**Table 4:** Nanoformulations with active targeting being studied in the clinics

Name	Formulation	Type	Targeting	Indications	Phase
BIND-014	PL(G)A-PEGylated Docataxel	Polymeric NP	PSMA specific receptor	Metastatic castration-resistant prostate cancer	Phase II (NCT01812746)
SP1049-C	Pluronic-b-copolymer doxorubicin		Pgp protein	Advanced adenocarcinoma of the esophagus	Phase II ([76])
MM-302	HER2-targeted PEGylated antibody-liposomal doxorubicin	Liposomal NP		HER2-positive metastatic cancer	Phase II (NCT02213744)
MCC-465	Liposomal Doxorubicin with F(ab’)2 fragment of GAH human Mab		GAH	Stomach cancer	Phase I ([77])
2B3-101	Doxorubicin with glutathione		Glutathione transporter	Brain metastasis Menongeal carcinomatosis	Phase I/IIa (NCT01386580) Phase II (NCT01818713
MBP-426	Oxaliplatin with transferrin		Transferrin receptor	Metastatic solid tumours	Phase I (NCT03002103)
anti-EGFR ILs-DOX	Doxorubicin-loaded Anti-EGFR immunoliposomes (C225-ILs-dox)		EGFR	High-grade gliomas	Phase I (NCT03603379)
IMMU-132	Trop-2 MAb and SN-38 conjugate	Drug - antibody conjugated	trophoblastic cell-surface antigen-2 (Trop-2)	Epithelial cancers	Phase I/II (NCT01631552)
SGN-35	MMAE coupled to CD30-targeted antibody		CD30 receptor	Relapsed or refractory Hodgkin lymphoma	Phase II (NCT00848926)

PSMA: prostate-specific membrane antigen; NPs: nanoparticles

Regarding drug conjugates, Sacituzumab-govitecan (IMMU-132) is a new antibody-drug conjugate targeting the human-trophoblast-cell-surface antigen 2 (Trop-2) conjugated with the active metabolite of irinotecan (SN-38). Trop-2 over-expression is related to invasiveness and poor prognosis in multiple human carcinomas. A phase II study in patients with uterine and ovarian carcinosarcomas is undergoing^[80]^. However, as promising as targeted therapies may seem, increasing the efficacy of the treatment is still challenging. An example is MM-302, a HER2-targeted PEGylated antibody-liposomal doxorubicin conjugate that targets HER2 over-expressing tumour cells. Although phase I results in HER2-positive metastatic cancer patients were promising, Phase II studies were stopped because they could not demonstrate the benefit over the current treatments with trastuzumab and pertuzumab^[81]^.

In comparison with the number of undergoing clinical trials, there are much more pre-clinical studies regarding targeted nanomedicines being reported. Available data in breast and colon cancer cell lines show that specific targeting can enhance the performance of nanomedicines and sensitizes CSC to paclitaxel based chemotherapy^[82]^. Another example is the use of the antibody CAB51 against human epithelial growth receptor 2 (HER2, ErbB2). It has been linked to cationic SLNs to evaluate the potential of targeting SLNs against breast cancer cells. The effect on MCF-7 and BT-474 cells showed a clearly increased level of NPs internalization^[83]^. Regarding functionalization with aptamers, there is a highly water-soluble nucleolin aptamer (NucA) paclitaxel conjugate that delivers PTX to the tumour site. NucA interacts with nucleolin protein which it is found expressed on the surface of cancer cells. Thus, NucA could be a promising tumour-targeting element for developing paclitaxel derivatives^[84]^. The most used glycoprotein for active targeting is transferrin because its receptors are known to be over-expressed in cancer cells surface. An example is the encapsulation of monomyristin, a monoacylglycerol that activates the intrinsic apoptotic mitochondrial pathway in HeLa cells, into dextran-covered polylactide (PLA) NPs functionalised with transferrin^[85]^.

As stated, combination therapy is a promising approach that can be enhanced with the utilization of specific targeting to overcome resistance of tumour cells. In one study, a biotin-/lactobionic acid modified PEG-PLGA-PEG copolymer with curcumin and 5-fluorouracil was synthesized to enhance the treatment of hepatocellular carcinoma. The dual-targeting and drug-loaded co-delivery nanosystem showed an increased cellular uptake and higher cytotoxicity of tumour cells. Therefore, dual-targeting strategies with co-delivery of therapeutics in a single nano-carrier can be used to achieve better intracellular delivery and synergistic anti-cancer efficacy^[86]^.

## Current drawbacks in bench to bed translation

Even though promising preclinical data regarding the use of targeted nanomedicines has been achieved, the clinical outcomes are still modest. There are several reasons for the limited clinical translation of targeted cancer nanomedicines. Some of them include the poor understanding of the biology (i.e., cellular and molecular understanding of the biological processes that will modulate NPs behaviour and fate *in vivo*), the interference of biological barriers, the misinterpretation of drug delivery concepts, a poor cost-effectiveness ratio, and a variety of manufacture and scale-up difficulties^[87]^. Besides, the engineering of a DDS with active targeting might increase the complexity and potential immunogenicity of the whole system. Also, it makes it more difficult, time consuming, and expensive to develop^[31]^. In addition, numerous limitations exist for clinical applications of some active targeting drugs because of their rapid elimination by the reticuloendothelial system and high tumour interstitial fluid pressure^[88]^. A lot of efforts are devoted to developing the “perfect NPs” with a wide range of specific ligands in their surface during preclinical development. It is fascinating what bioengineering is capable to achieve and all the complexity that can be designed and synthesized in a single platform. However, over the last years it has been demonstrated that translation to clinical use gets poorer when complexity gets richer. Up to date, the most clinically successful NP is Abraxane. This albumin-bound paclitaxel NP is a simply engineered NP; however, it is very elegant since albumin is recognized by gp60 receptors of the endothelial cells that guide the extravasation of Abraxane^[89]^. Many methods of functionalization are published, but most of them lack reproducibility. The functionalization process is very complicated and requires different conditions for each efficient surface modification. It involves a multi-step processing to formulate complex targeted NPs which in turn compromises final production yields. Also, it is important to not forget that even the exhaustive processing for the synthesis of NPs, obtaining uniform size is not yet a reality^[73]^. Due to the process variation there is a lack of scalability that prevents large-scale manufacturing^[90]^. It is important to develop highly reproducible single step methods to functionalize NP surface^[73]^. Further complication is presented by the biological conditions of the tumour. Even though the presence of the EPR effect, for certain NPs to reach and enter the TME may be a considerable challenge. Even though the NP extravasate into the tumour vicinity, targeted NPs commonly bind cells with high affinity in the outer layer of the TME. Thus, they cannot easily penetrate to the inner parts of the tumour^[31]^. This phenomenon is known as the binding site barrier (BSB) which prevents deeper penetration of NPs into the tumours. Specifically, the BSB limits NP diffusion trough the TME and results in unintended internalization of NPs by stromal cells located near blood vessels. The major components of the BSB are tumour activated fibroblasts. The proximity to the blood vessels and the expression of protein receptors also complicate the penetration of NPs into tumours^[91]^. Tumour activated fibroblasts need to be considered to overcome the BSB and be able to reach the desired cells in the TME. In addition, TME is also crucial as a player of treatment resistance of many cancers. Moreover, the best strategy to prevent tumour remission should be the elimination of all aggressive cells within the tumour^[24]^. This could be achieved by the combination of various therapeutic molecules and a combination of gene therapy approaches.

Further, there is an actual unmet need to synergize passive and active targeting to improve the accumulation of nanoparticles at the desired site while at the same time enhancing their intracellular penetration^[69]^. Due to the tumour tissue barriers and tumour heterogeneity, there is an overestimated EPR effect in clinical tumour therapy. Besides, there is the existence of elevated tumour interstitial fluid pressure that reduces drug delivery efficacy, thus limiting NP distribution into the TME. The increased interstitial fluid pressure has been reported in many solid tumours, such as breast, colorectal cancers, and melanoma^[88,92]^. Yet, the BSB and the interstitial fluid pressure have not been completely taken into account in preclinical studies due to the existence of discrepancies between animal models and human tumours^[77]^. The use of murine models in preclinical studies to assess the penetration of NPs into the tumour has poor translation, since tiny murine tumours differ substantially in size and pathophysiology regarding human tumours. In this context, it is well known that immune-mediated adverse effects might appear after a nanoformulation is administered, since many clinically relevant side effects have been reported^[93]^. The extended use of immunosuppressed mice models might hamper the study of how the immune system might interfere with NPs. In this regard, the more common toxicities linked to NP failure include: erythrocyte damage, thrombogenicity (platelet aggregation, plasma coagulation), cytokine-mediated inflammation and cytokine storming, pyrogenicity, and anaphylaxis and other complement activation mediated reactions, as well as recognition and uptake by the cells of the MPS^[94]^.

In fact, there is a wide variety of nanomaterials available and their physicochemical properties (i.e., size, biocompatibility, and surface chemistry) play an important role in the activation of an immune response. Moreover, the introduction of a targeting ligand changes the properties of the NPs and often makes them even more difficult to pass unnoticed. One of the grand challenges in the NP characterization is screening for immunotoxicities. These studies are based on the estimation of immunoreactive contaminants, such as excipients and linkers^[94]^. Although there are current standard methods, they are insufficient to address the broad spectrum of biomarkers that indicate NP immunotoxicity. In addition, there is an absence of consensus on well characterized reference materials. Therefore, preclinical studies often depend on nanomedicines with known clinical immunotoxicities (e.g., Doxil for complement activation and anaphylaxis)^[94]^. Again, the use of immunosuppressed animals makes it even more difficult to determine immunotoxicities related to NPs. Similarly, in clinical studies, patients are premedicated with immunosuppressors to prevent adverse reactions. Screening for these toxicities in preclinical development would help to prevent potentially toxic formulations^[94]^. However, currently, the properties of nanoparticles (particularly, targeted ones) have not yet been fully exploited.

Investment in nanomedicine in the early 2000s accelerated the development of nanoformulations that are currently available in the market. However, even with the sales success of some of them (e.g., Abraxane), there are financial challenges that hamper the development of new nanoformulations. As it has been previously explained, it is not easy to demonstrate improved efficacy and safety compared to other validated and marketed products for the same indication. Indeed, the majority of approved nanodrugs are based on currently approved drugs which faces reduced financial risk because the efficacy and safety of the active ingredient had already been established^[52]^. On the contrary, more complex economic considerations are involved when developing a nanodrug that contains a new chemical entity. Moreover, the complexity to design a specific ligand and the conjugation techniques make the whole process more expensive with a difficult scale-up. The cost of using complex chemistry, controlled quality manufacturing, and scaled production is elevated^[52]^. In addition, the lack of specific general protocols for the study of safety or efficacy of these nanomedicines is hampering clinical development. Apart from all the high costs involved, regulatory requirements also make market entry difficult. Various regulatory agencies like EMA and FDA started their discussion on the classification of nanomaterial and how to regulate them to ensure proper efficacy and safety of these materials. For example, the regulatory system in Europe allows the marketing authorization applicants to receive scientific counselling during early stages of research and development. Also, the Nanotechnology Characterization Laboratory at the National Cancer Institute in the US collects all the data on nanomedicines in oncology. Indeed, integration between materials and translational issues, such as more appropriate disease models, are essential for developing accurate regulation of nanomedicines^[90]^. Actual mice models used in cancer nanomedicine present some drawbacks in terms of physiopathological properties, as mentioned in the previous chapter. The use of patient derived xenografts (PDX) is a promising alternative to better mimic human TME and study the accumulation and extravasation of NPs as well as specific targeting in a more real-life environment. However, the need to use immune-supressed models is again a drawback because mice lacking an immune system hamper the study of the interactions among NPs, cancer cells, and immune cells in the TME^[95]^. Still, in order to represent the heterogeneity of human tumour and the drug sensitivity ranges, it is important to create a large bank of PDX of different tumour types^[96]^. Recently, immunodeficient mice engrafted with human immune systems have been established and are a powerful tool for the next generation of PDX models. The immune deficient mice are irradiated by gamma irradiation and then, human hematopoietic stem cells (HSCs) are introduced in the immune deficient mice to obtain a humanised mouse model. Finally, with the insertion of small parts of the human tumour the result is a PDX model with a human immune system. For example, it has already been reported, a triple negative breast cancer PDX model with humanized mice that provides evidence that supports its use for the pre-clinical investigation of immune-based therapies. Unfortunately, currently expensive production costs of these models limits their wider use^[95]^.

### Future perspectives of nanomedicine

All mentioned drawbacks translate into poor clinical outcomes, particularly of targeted therapies. Thus, there is a clear need to focus on existing nano-carriers, combination therapies, patient selection, and ways to enable rapid and more efficient clinical translation^[97]^. This poor clinical translation is also seen in the total number of clinical trials (only 2%) in comparison with the total number of publications in the field of cancer nanomedicine. It is necessary to promote the entrance of new products in clinical phases. Many alternatives have been screened to provide a safer and a better DDS treatment. Hydrogels are three-dimensional networks formed by hydrophilic polymer chains build in a water-rich environment which possess a broadly tuneable physical and chemical properties. They are formed through the cross-linking of hydrophilic polymer chains. The water-rich nature of hydrogels makes them widely applicable, including for drug delivery^[98]^. Their advantage against active and passive nanomedicines is that they can provide localized and targeted therapy regardless of the blood supply and microvasculature morphology of the tumour. Also a hydrogel-based system could extend the physical stability of chemotherapeutic agents or nucleic acids for months^[88]^. In addition, hydrogels can deliver two or more therapeutic agents in the same platform in a sustained manner. Co-delivery of multiple therapeutic agents with different targets is known to be a promising strategy to overcome drug resistance and reduce the chance of metastatic progression. Recently, paclitaxel and lapatinib NP in a thermosensitive hydrogel showed a synergistic effect^[99]^. Also, RNAi-chemotherapeutic drug combinations can effectively overcome tumour resistance to chemotherapeutic agents by inhibiting the mentioned multidrug resistance pathways. For example, protein kinase B (AKT)-targeted gene therapy along with paclitaxel given as linoleic acid-coupled pluronic hydrogel showed possible synergistic anti-cancer effects by downregulation of AKT signalling and facilitation of apoptosis induction^[100]^.

Research should continue to explore new materials to improve DDS for anti-cancer therapies. An interesting alternative is to learn more about nature’s own delivery systems. In this regard, exosomes are a class of extracellular vesicles that contain proteins, nucleic acids, and lipids. They are the key players of numerous biological processes both pathologic and non-pathologic^[101]^. In cancer development, exosomes are further described as mediators of tumour-stroma interaction known as tumour-derived exosomes (TDEs). This crosstalk is known to be involved in various pathophysiological processes including migration, treatment resistance and metastasis. TDEs have the capacity to induce EMT and enhance migratory activity. This was observed in glioblastoma cell lines, lung carcinoma cells, and a model of gastric cancer *in vivo*^[102]^. This communication may confer epigenetic changes in the neighbouring cells by transportation of miRNAs. For example, exosomal miR-23a supports the EMT-promoting effect by inhibiting E-cadherin synthesis in lung carcinoma and melanoma cells. TDEs from tumour cells that have undergone EMT can in turn stimulate neighbouring cells to acquire EMT like features^[103]^. This may explain the communication in the TME between DDC and CSC to undergo conversion and maintain a dynamic phenotype. In addition, the miRNA secreted by TDEs influence cell invasion and intravasation to blood vessels. For instance, exosomal miR-105 in the serum of patients with breast cancer is a prognostic marker for the later development of metastasis^[104]^. Exosomes can also transport classical chemotherapeutics (e.g., Doxorubicin and Cisplatin) to the tumour cells with a reduction of toxicity, such as the one reported in drug-resistant lung cancer cells^[105]^. Moreover, exosomes can also transport nucleic acids and be used as a vector for gene delivery purposes to the tumour cells^[101]^. Also, they can be further engineered to present on their membrane targeting ligands to improve biodistribution^[106]^. The metastatic location is not chosen randomly, instead it is rather a consequence of a tumour-stroma interaction in the host organ (organotropic metastasis). It has been reported that metastatic cancer cells derived from a particular tumour site present enhanced metastatic ability to specific organs, independent of the anatomy of blood and lymphatic vessels that drain the primary tumour site^[103]^. There are many questions regarding how exosomes are directed to specific metastatic organs enabling organotrophic metastatic growth. For example, exosomes from breast cancer cells move to lung tissue in mouse models. Exosome biodistribution matched the organotropic metastatic spread *in vitro* in cell lines from various types of cancer such as breast and pancreatic cancer^[103]^. This opens a new therapeutic window to target metastasis, which has not been achieved with conventional nanosystems. Besides, natural DDS seem to be unnoticed by the immune system. The large quantity of exosomes (1.5 billion exosomes per mL) in human blood allows their use as anti-cancer therapies. Nevertheless, they have not yet reached the clinics. Lack of standard isolation and loading protocols^[103]^ and important drawbacks regarding scale-up production, are still unsolved problems.

## Declarations

### Copyright

© The Author(s) 2021.

## References

[1] https://gco.iarc.fr/today/data/factsheets/populations/908-europe-fact-sheets.pdf.

[2] Ferlay J, Colombet M, Soerjomataram I, Dyba T, Randi G (2018). Cancer incidence and mortality patterns in Europe: estimates for 40 countries and 25 major cancers in 2018.. Eur J Cancer.

[3] Wang X, Zhang H, Chen X (2019). Drug resistance and combating drug resistance in cancer.. Cancer Drug Resist.

[4] Mansoori B, Mohammadi A, Davudian S, Shirjang S, Baradaran B (2017). The different mechanisms of cancer drug resistance : a brief review.. Tabriz Univ Med Sci.

[5] Longacre M, Snyder N, Sarkar S (2014). Drug resistance in cancer : an overview.. Cancers (Basel).

[6] Xue X, Liang XJ (2012). Overcoming drug efflux-based multidrug resistance in cancer with nanotechnology.. Chin J Cancer.

[7] Robey RW, Pluchino KM, Hall MD, Fojo AT, Bates SE, Gottesman MM (2018). Revisiting the role of efflux pumps in multidrug-resistant cancer.. Nat Rev Cancer.

[8] Gener P, Rafael DF, Fernández Y (2016). Cancer stem cells and personalized cancer nanomedicine.. Nanomedicine (Lond).

[9] Lepeltier E, Rijo P, Rizzolio F (2020). Nanomedicine to target multidrug resistant tumors.. Drug Resist Updat.

[10] Allocati N, Masulli M, Di Ilio C, Federici L (2018). Glutathione transferases: substrates, inihibitors and pro-drugs in cancer and neurodegenerative diseases.. Oncogenesis.

[11] Westover D, Zugazagoitia J, Cho BC, Lovly CM, Paz-Ares L (2018). Mechanisms of acquired resistance to first- and second-generation EGFR tyrosine kinase inhibitors.. Ann Oncol.

[12] Wu S, Shih J (2018). Management of acquired resistance to EGFR TKI - targeted therapy in advanced non-small cell lung cancer.. Mol Cancer.

[13] Wang S, Song Y, Liu D (2017). EAI045: the fourth-generation EGFR inhibitor overcoming T790M and C797S resistance.. Cancer Lett.

[14] Desai A, Yan Y, Gerson SL (2018). Advances in therapeutic targeting of the DNA damage response in cancer.. DNA Repair (Amst).

[15] Hosoya N, Miyagawa K (2014). Targeting DNA damage response in cancer therapy.. Cancer Sci.

[16] Li D, Yang L (2018). Olaparib nanoparticles potentiated radiosensitization effects on lung cancer.. Int J Nanomedicine.

[17] Mohammad RM, Muqbil I, Lowe L (2015). Broad targeting of resistance to apoptosis in cancer,. Semin Cancer Biol.

[18] Zhou Y, Huang F, Yang Y (2018). Paraptosis-inducing nanomedicine overcomes cancer drug resistance for a potent cancer therapy.. Small.

[19] Sepand MR, Ranjbar S, Kempson IM (2020). Targeting non-apoptotic cell death in cancer treatment by nanomaterials: Recent advances and future outlook.. Nanomedicine.

[20] Roberti A, Valdes AF, Torrecillas R, Fraga MF, Fernandez AF (2019). Epigenetics in cancer therapy and nanomedicine.. Clin Epigenetics.

[21] Fardi M, Solali S, Farshdousti Hagh M (2018). Epigenetic mechanisms as a new approach in cancer treatment: an updated review.. Genes Dis.

[22] Takeshima H, Ushijima T (2019). Accumulation of genetic and epigenetic alterations in normal cells and cancer risk.. NPJ Precis Oncol.

[23] Cheng Y, He C, Wang M (2019). Targeting epigenetic regulators for cancer therapy: mechanisms and advances in clinical trials.. Signal Transduct Target Ther.

[24] Gener P, Seras-franzoso J, Callejo PG (2018). Review article dynamism, sensitivity, and consequences of mesenchymal and stem-like phenotype of cancer cells.. Stem Cells Int.

[25] LeBleu VS, Kalluri R (2018). A peek into cancer-associated fibroblasts: origins, functions and translational impact.. Dis Model Mech.

[26] Roma-Rodrigues C, Mendes R, Baptista PV, Fernandes AR (2019). Targeting Tumor Microenvironment for Cancer Therapy.. Int J Mol Sci.

[27] Tomita H, Tanaka K, Tanaka T, Hara A (2016). Aldehyde dehydrogenase 1A1 in stem cells and cancer.. Oncotarget.

[28] Gener P, Callejo PG, Seras-Franzoso J (2020). The potential of nanomedicine to alter cancer stem cell dynamics: the impact of extracellular vesicles.. Nanomedicine (Lond).

[29] Yu AM, Jian C, Yu AH, Tu MJ (2019). RNA therapy: are we using the right molecules?. Pharmacol Ther.

[30] Gómez-Aguado I, Rodríguez-Castejón J, Vicente-Pascual M, Rodríguez-Gascón A, Solinís MÁ, Del Pozo-Rodríguez A (2020). Nanomedicines to deliver mRNA: state of the art and future perspectives.. Nanomaterials (Basel).

[31] Vaughan HJ, Green JJ, Tzeng SY (2020). Cancer-targeting nanoparticles for combinatorial nucleic acid delivery.. Adv Mater.

[32] Rafael D, Gener P, Andrade F (2018). AKT2 siRNA delivery with amphiphilic-based polymeric micelles show efficacy against cancer stem cells.. Drug Deliv.

[33] Gener P, Rafael D, Seras-Franzoso J (2019). Pivotal Role of AKT2 during Dynamic Phenotypic Change of Breast Cancer Stem Cells.. Cancers (Basel).

[34] Davis ME, Zuckerman JE, Choi CH (2010). Evidence of RNAi in humans from systemically administered siRNA via targeted nanoparticles.. Nature.

[35] Pearce AK, O’Reilly RK (2019). Insights into Active Targeting of Nanoparticles in Drug Delivery: Advances in Clinical Studies and Design Considerations for Cancer Nanomedicine.. Bioconjug Chem.

[36] Tabernero J, Shapiro GI, LoRusso PM (2013). First-in-humans trial of an RNA interference therapeutic targeting VEGF and KSP in cancer patients with liver involvement.. Cancer Discov.

[37] Schultheis B, Strumberg D, Santel A (2014). First-in-human phase I study of the liposomal RNA interference therapeutic Atu027 in patients with advanced solid tumors.. J Clin Oncol.

[38] Wagner MJ, Mitra R, McArthur MJ (2017). Preclinical Mammalian Safety Studies of EPHARNA (DOPC Nanoliposomal EphA2-Targeted siRNA).. Mol Cancer Ther.

[39] Demeure MJ, Armaghany T, Ejadi S (2016). A phase I/II study of TKM-080301, a *PLK1* -targeted RNAi in patients with adrenocortical cancer (ACC).. JCO.

[40] Jo DH, Kim JH, Lee TG, Kim JH (2015). Size, surface charge, and shape determine therapeutic effects of nanoparticles on brain and retinal diseases.. Nanomedicine.

[41] Wicki A, Witzigmann D, Balasubramanian V, Huwyler J (2015). Nanomedicine in cancer therapy: challenges, opportunities, and clinical applications.. J Control Release.

[42] https://www.researchgate.net/publication/312334531_An_Overview_of_Nanoparticle_Biocompatibility_for_Their_Use_in_Nanomedicine_Innovation_and_Production.

[43] Mishra P, Nayak B, Dey R (2016). PEGylation in anti-cancer therapy: an overview.. Asian J Pharm Sci.

[44] Tran S, DeGiovanni PJ, Piel B, Rai P (2017). Cancer nanomedicine: a review of recent success in drug delivery.. Clin Transl Med.

[45] Fülöp T, Kozma GT, Vashegyi I (2019). Liposome-induced hypersensitivity reactions: Risk reduction by design of safe infusion protocols in pigs.. J Control Release.

[46] Campos J, Severino P, Santini A (2020). Solid lipid nanoparticles (SLN). Nanopharmaceuticals..

[47] Bakhtiary Z, Barar J, Aghanejad A (2017). Microparticles containing erlotinib-loaded solid lipid nanoparticles for treatment of non-small cell lung cancer.. Drug Dev Ind Pharm.

[48] Soni N, Soni N, Pandey H, Maheshwari R, Kesharwani P, Tekade RK (2016). Augmented delivery of gemcitabine in lung cancer cells exploring mannose anchored solid lipid nanoparticles.. J Colloid Interface Sci.

[49] Gener P, Montero S, Xandri-Monje H (2020). Zileuton™ loaded in polymer micelles effectively reduce breast cancer circulating tumor cells and intratumoral cancer stem cells.. Nanomedicine.

[50] Yu X, Di Y, Xie C (2015). An in vitro and in vivo study of gemcitabine-loaded albumin nanoparticles in a pancreatic cancer cell line.. Int J Nanomedicine.

[51] Zhou Y, Yang J, Rhim JS, Kopeček J (2013). HPMA copolymer-based combination therapy toxic to both prostate cancer stem/progenitor cells and differentiated cells induces durable anti-tumor effects.. J Control Release.

[52] Ventola CL (2017). Progress in nanomedicine: approved and investigational nanodrugs progress in nanomedicine.. Pharmacol Ther.

[53] Li Y, Zhang H (2019). Nanoparticle-based drug delivery systems for enhanced tumor-targeting treatment.. J Biomed Nanotechnol.

[54] Watermann A, Brieger J (2017). Mesoporous Silica nanoparticles as drug delivery vehicles in cancer.. Nanomaterials (Basel).

[55] Gabizon A, Catane R, Uziely B (1994). Prolonged circulation time and enhanced accumulation in malignant exudates of doxorubicin encapsulated in polyethylene-glycol coated liposomes 1.. Cancer Res.

[56] Miele E, Spinelli GP, Miele E, Tomao F, Tomao S (2009). Albumin-bound formulation of paclitaxel (Abraxane ® ABI-007) in the treatment of breast cancer.. Int J Nanomedicine.

[57] Hamad I, Moghimi SM (2008). Critical issues in site-specific targeting of solid tumours: the carrier, the tumour barriers and the bioavailable drug.. Expert Opin Drug Deliv.

[58] Heo YA, Syed YY, Keam SJ (2019). Pegaspargase: a review in acute lymphoblastic leukaemia.. Drugs.

[59] Choi YH, Han H (2018). Nanomedicines: current status and future perspectives in aspect of drug delivery and pharmacokinetics.. J Pharm Invest.

[60] Louie AC (2019). CPX-351: a nanoscale liposomal co-formulation of daunorubicin and cytarabine with unique biodistribution and tumor cell uptake properties.. Int J Nanomedicine.

[61] Liu B, Chen Z (2017). Co-delivery of paclitaxel and TOS-cisplatin via TAT-targeted solid lipid nanoparticles with synergistic antitumor activity against cervical cancer.. Int J Nanomedicine.

[62] Katiyar SS, Muntimadugu E, Rafeeqi TA, Domb AJ, Khan W (2016). Co-delivery of rapamycin- and piperine-loaded polymeric nanoparticles for breast cancer treatment.. Drug Deliv.

[63] Khaledi S, Jafari S, Hamidi S, Molavi O, Davaran S (2020). Preparation and characterization of PLGA-PEG-PLGA polymeric nanoparticles for co-delivery of 5-Fluorouracil and Chrysin.. J Biomater Sci Polym Ed.

[64] Gabizon AA, Tahover E, Golan T (2020). Pharmacokinetics of mitomycin-c lipidic prodrug entrapped in liposomes and clinical correlations in metastatic colorectal cancer patients.. Invest New Drugs.

[65] Chao J, Lin J, Frankel P (2017). Pilot trial of CRLX101 in patients with advanced, chemotherapy-refractory gastroesophageal cancer.. J Gastrointest Oncol.

[66] Golombek SK, May J, Theek B, Appold L (2018). Tumor targeting via EPR: strategies to enhance patient responses.. Adv Drug Deliv Rev.

[67] Islam W, Fang J, Imamura T (2018). Augmentation of the enhanced permeability and retention effect with nitric oxide-generating agents improves the therapeutic effects of nanomedicines.. Mol Cancer Ther.

[68] Azzopardi EA, Ferguson EL, Thomas DW (2013). The enhanced permeability retention effect: a new paradigm for drug targeting in infection.. J Antimicrob Chemother.

[69] Xu X, Ho W, Zhang X (2016). Cancer nanomedicine: from targeted delivery to combination therapy.. Trends Mol Med.

[70] Salvioni L, Rizzuto MA, Bertolini JA, Pandolfi L, Colombo M, Prosperi D (2019). Thirty years of cancer nanomedicine: success, frustration, and hope.. Cancers (Basel).

[71] Adamo G, Campora S, Ghersi G (2017). Chapter 3 - functionalization of nanoparticles in specific targeting and mechanism release..

[72] Subbiah R, Veerapandian M, Yun KS (2010). Nanoparticles: functionalization and multifunctional applications in biomedical sciences.. Curr Med Chem.

[73] Thiruppathi R, Mishra S, Ganapathy M, Padmanabhan P, Gulyás B (2017). Nanoparticle functionalization and its potentials for molecular imaging.. Adv Sci (Weinh).

[74] Mout R, Moyano DF, Rana S, Rotello VM (2012). Surface functionalization of nanoparticles for nanomedicine.. Chem Soc Rev.

[75] Gonda A, Zhao N, Shah JV (2019). Engineering tumor-targeting nanoparticles as vehicles for precision nanomedicine.. Med One.

[76] Valle JW, Armstrong A, Newman C (2011). A phase 2 study of SP1049C, doxorubicin in P-glycoprotein-targeting pluronics, in patients with advanced adenocarcinoma of the esophagus and gastroesophageal junction.. Invest New Drugs.

[77] Attia MF, Anton N, Wallyn J, Omran Z, Vandamme TF (2019). An overview of active and passive targeting strategies to improve the nanocarriers efficiency to tumour sites.. J Pharm Pharmacol.

[78] Zununi Vahed S, Fathi N, Samiei M, Maleki Dizaj S, Sharifi S (2019). Targeted cancer drug delivery with aptamer-functionalized polymeric nanoparticles.. J Drug Target.

[79] Autio KA, Dreicer R, Anderson J (2018). Safety and Efficacy of BIND-014, a Docetaxel Nanoparticle Targeting Prostate-Specific Membrane Antigen for Patients With Metastatic Castration-Resistant Prostate Cancer: A Phase 2 Clinical Trial.. JAMA Oncol.

[80] Lopez S, Perrone E, Bellone S (2020). Preclinical activity of sacituzumab govitecan (IMMU-132 ) in uterine and ovarian carcinosarcomas.. Oncotarget.

[81] Munster P, Krop IE, LoRusso P (2018). Safety and pharmacokinetics of MM-302, a HER2-targeted antibody-liposomal doxorubicin conjugate, in patients with advanced HER2-positive breast cancer: a phase 1 dose-escalation study.. Br J Cancer.

[82] Gener P, Gouveia LP, Sabat GR (2015). Fluorescent CSC models evidence that targeted nanomedicines improve treatment sensitivity of breast and colon cancer stem cells.. Nanomedicine.

[83] Souto EB, Doktorovova S, Campos JR, Martins-Lopes P, Silva AM (2019). Surface-tailored anti-HER2/neu-solid lipid nanoparticles for site-specific targeting MCF-7 and BT-474 breast cancer cells.. Eur J Pharm Sci.

[84] Li F, Lu J, Liu J (2017). A water-soluble nucleolin aptamer-paclitaxel conjugate for tumor-specific targeting in ovarian cancer.. Nat Commun.

[85] Boondireke S, Léonard M, Durand A, Thanomsub Wongsatayanon B (2019). Encapsulation of monomyristin into polymeric nanoparticles improved its in vitro antiproliferative activity against cervical cancer cells.. Colloids Surf B Biointerfaces.

[86] Ni W, Li Z, Liu Z (2019). Dual-targeting nanoparticles: codelivery of curcumin and 5-fluorouracil for synergistic treatment of hepatocarcinoma.. J Pharm Sci.

[87] van der Meel R, Lammers T, Hennink WE (2017). Cancer nanomedicines: oversold or underappreciated?. Expert Opin Drug Deliv.

[88] Sepantafar M, Maheronnaghsh R, Mohammadi H (2017). Engineered hydrogels in cancer therapy and diagnosis.. Trends Biotechnol.

[89] Villaverde G, Baeza A (2019). Targeting strategies for improving the efficacy of nanomedicine in oncology.. Beilstein J Nanotechnol.

[90] Tambe V, Maheshwari R, Chourasiya Y, Choudhury H, Gorain B, Tekade RK (2019). Clinical aspects and regulatory requirements for nanomedicines. Basic Fundamentals of Drug Delivery..

[91] Miao L, Newby JM, Lin CM (2016). The binding site barrier elicited by tumor associated fibroblasts interferes disposition of nanoparticles in the stroma-vessel type tumors.. ACS Nano.

[92] Libutti SK, Tamarkin L, Nilubol N (2018). Targeting the invincible barrier for drug delivery in solid cancers: interstitial fluid pressure.. Oncotarget.

[93] Szebeni J, Simberg D, González-Fernández Á, Barenholz Y, Dobrovolskaia MA (2018). Roadmap and strategy for overcoming infusion reactions to nanomedicines.. Nat Nanotechnol.

[94] Dobrovolskaia MA, Shurin M, Shvedova AA (2016). Current understanding of interactions between nanoparticles and the immune system.. Toxicol Appl Pharmacol.

[95] Murayama T, Gotoh N (2019). Patient-derived xenograft models of breast cancer and their application.. Cells.

[96] Gazdar AF, Hirsch FR, Minna JD (2016). From mice to men and back: an assessment of preclinical model systems for the study of lung cancers.. J Thorac Oncol.

[97] Lammers T, Kiessling F, Ashford M, Hennink W, Crommelin D, Storm G (2016). Cancer nanomedicine: Is targeting our target?. Nat Rev Mater.

[98] Zhang YS, Khademhosseini A (2017). Advances in engineering hydrogels.. Science.

[99] Hu H, Lin Z, He B (2015). A novel localized co-delivery system with lapatinib microparticles and paclitaxel nanoparticles in a peritumorally injectable in situ hydrogel.. J Control Release.

[100] Guo DD, Hong SH, Jiang HL (2012). Synergistic effects of Akt1 shRNA and paclitaxel-incorporated conjugated linoleic acid-coupled poloxamer thermosensitive hydrogel on breast cancer.. Biomaterials.

[101] Ruivo CF, Adem B, Silva M, Melo SA (2017). The biology of cancer exosomes: insights and new perspectives.. Cancer Res.

[102] Steinbichler TB, Dudás J, Riechelmann H, Skvortsova II (2017). The role of exosomes in cancer metastasis.. Semin Cancer Biol.

[103] Kim J, Kim TY, Lee MS, Mun JY, Ihm C, Kim SA (2016). Exosome cargo reflects TGF-β1-mediated epithelial-to-mesenchymal transition (EMT) status in A549 human lung adenocarcinoma cells.. Biochem Biophys Res Commun.

[104] Kosaka N (2016). Decoding the secret of cancer by means of extracellular vesicles.. J Clin Med.

[105] Kim MS, Haney MJ, Zhao Y (2016). Development of exosome-encapsulated paclitaxel to overcome MDR in cancer cells.. Nanomedicine.

[106] Walker S, Busatto S, Pham A (2019). Extracellular vesicle-based drug delivery systems for cancer treatment.. Theranostics.

